# Sono-Electro-Magnetic Therapy for Treating Chronic Pelvic Pain Syndrome in Men: A Randomized, Placebo-Controlled, Double-Blind Trial

**DOI:** 10.1371/journal.pone.0113368

**Published:** 2014-12-29

**Authors:** Thomas M. Kessler, Livio Mordasini, Christian Weisstanner, Peter Jüni, Bruno R. da Costa, Roland Wiest, George N. Thalmann

**Affiliations:** 1 Department of Urology, University of Bern, Bern, Switzerland; 2 Neuro-Urology, Spinal Cord Injury Center & Research, University of Zürich, Balgrist University Hospital, Zürich, Switzerland; 3 Support Center for Advanced Neuroimaging (SCAN), University Institute of Diagnostic and Interventional Neuroradiology, Inselspital, University of Bern, Bern, Switzerland; 4 Institute of Social and Preventive Medicine and Clinical Trials Unit, University of Bern, Bern, Switzerland; Eberhard-Karls University, Germany

## Abstract

**Objective:**

To assess the efficacy and safety of sono-electro-magnetic therapy compared to placebo in men with refractory CPPS.

**Patients and Methods:**

In a randomized, placebo-controlled, double-blind single center trial, we assessed the effect of sono-electro-magnetic therapy in men with treatment refractory CPPS. Sixty male patients were randomly assigned to treatment with either sono-electro-magnetic (n = 30) or placebo therapy (n = 30) for 12 weeks. The primary outcome was a change in the National Institutes of Health Chronic Prostatitis Symptom Index (NIH-CPSI) from baseline to 12 weeks.

**Results:**

The 12-week difference between sono-electro-magnetic and placebo therapy in changes of the NIH-CPSI total score was −3.1 points (95% CI −6.8 to 0.6, p = 0.11). In secondary comparisons of NIH-CPSI sub-scores, we found differences between groups most pronounced for the quality-of-life sub-score (difference at 12 weeks −1.6, 95% CI −2.8 to −0.4, p = 0.015). In stratified analyses, the benefit of sono-electro-magnetic therapy appeared more pronounced among patients who had a symptom duration of 12 months or less (difference in NIH-CPSI total score −8.3, 95% CI −14.5 to 2.6) than in patients with a longer symptom duration (−0.8, 95% CI −4.6 to 3.1; p for interaction = 0.023).

**Conclusions:**

Sono-electro-magnetic therapy did not result in a significant improvement of symptoms in the overall cohort of treatment refractory CPPS patients compared to placebo treatment. Subgroup analysis indicates, however, that patients with a symptom-duration of 12 months or less may benefit from sono-electro-magnetic therapy, warranting larger randomized controlled trials in this subpopulation.

**Trial Registration:**

ClinicalTrials.gov NCT00688506

## Introduction

Male chronic pelvic pain syndrome (CPPS), also called chronic prostatitis, is a disabling disorder characterized by a non-malignant pain in the pelvic region that lasts for at least three months without evidence of infection or other obvious pathology [1]. Approximately 6%–12% of men suffer from CPPS; the disease affects millions worldwide [2]. It is similar to congestive heart failure, Crohn's disease, diabetes mellitus or angina [3] in lowering quality of life, and represents a serious economic burden on any health care system. Associated costs match those reported for peripheral neuropathy, low back pain, fibromyalgia, and rheumatoid arthritis [4].

Factors contributing to CPPS may include chemical irritants, pelvic floor muscle irregularities, and immunological, endocrine and neurological dysfunction. Presumptive triggers are many, and therapeutic approaches are both varied, and of limited effect [5]. Multimodal therapies are more successful than single therapies, and a combination of antibiotics, alpha-blockers and/or anti-inflammatories seems to help some patients [6], [7]. However, approximately 20% to 65% of all patients find no relief in conventional therapies [6], [8] and treatment of CPPS remains a challenge. Effective, easy to tolerate therapeutic alternatives are urgently needed.

Neuromodulative techniques, based on the theory of pain sensitization via descending and ascending pathways, have become increasingly popular for treating chronic pain, and have had promising results [9]–[11]. A pilot study that treated patients using a combination of different kinds of neuromodulation reported encouraging findings [12]. Thus, we performed a single-center, randomized, placebo-controlled, double-blind trial to assess the effect of the non-invasive sono-electro-magnetic therapy for treating men with refractory CPPS in reducing symptoms as measured by the National Institutes of Health Chronic Prostatitis Symptom Index (NIH-CPSI).

## Patients and Methods

### 2.1. Patients

Potential trial participants were recruited between November 2009 and July 2012 from the urological outpatient clinic at Bern University Hospital, Switzerland. In accordance with the EAU guidelines [1], all patients with CPPS included in the trial complained about pain perceived in pelvic structures for at least three months, without evidence of infection or other obvious pathologies. All patients considered for enrolment had complete urological evaluation, including duration of symptoms, physical examination, urinalysis, determination of prostate-specific antigen (PSA), free uroflowmetry and post void residual measurement. Included patients had been unsuccessfully treated with stepwise, multimodal therapy including the administration of doxycycline (treatment duration of at least four weeks in patients and two weeks in sexual partners), non-steroidal anti-inflammatory drugs and alpha-blocker therapy for at least six weeks. Patients had to have a NIH-CPSI total score ≥15 and NIH-CPSI pain sub-score ≥8. Exclusion criteria were chronic bacterial prostatitis (based on Meares-Stamey 3-glass test and post-prostatic massage urine culture), urinary tract infection (presence of bacteria in urinalysis), post void residual>100 mL, prostate cancer, urethral stricture, and age <18 years.

### 2.2. Trial design, treatment and follow-up

The protocol for this trial and supporting CONSORT checklist are available as supporting information; see S1 CONSORT Checklist and S1 Protocol. After the baseline examination, patients were randomly allocated to sono-electro-magnetic or placebo therapy based on computer-generated random numbers with a randomization ratio of 1∶1 and a block size of 60. The manufacturer pre-packed and sequentially numbered the active and placebo devices according to the concealed randomization schedule. Active and placebo devices were both produced by the same manufacturer. They looked identical, were packed identically, and the placebo device lit the same buttons when charged and when switched on as the verum device, but did not provide stimulation. Study nurses handed over the closed packs in sequential order and instructed patients on the use of the device. Patients, recruiting investigators, study nurses and physicians performing follow-up assessments were all unaware of the allocated treatment. Patients performed sono-electro-magnetic and placebo therapy at home, using the portable Sonodyn device (Sonodyn Corporation AG, Solothurn, Switzerland). They used a gel and applied the device on the perineum daily in the morning and evening, for the duration of ten minutes each time. Considering that there are no high-evidence level studies on the ideal neuromodulation parameters in CPPS, the devices for sono-electro-magnetic therapy were set to the manufacturer's recommended and preset stimulation parameters for treating musculoskeletal pain: ultrasound intensity of <100 mW/cm2 with an ultrasonic power of 12 mW and a frequency of 1.9 MHz, electric field force of <3 V/m and magnetic field force of 0.4 A/m. Patients could not see the settings and could not perceive the device working because it used subsensory stimulation.

Follow-up assessments were performed at 6 and 12 weeks, with an extended follow-up at 16 weeks. At each time-point, patients received a urological examination, NIH-CPSI, free uroflowmetry, post void residual, urinalysis and PSA measurement. During the 12-week visit, therapy was halted and the stimulation device was collected. Adherence to treatment was assessed using a patient diary, requiring patients to record daily whether they had used the device in the morning and evening.

### 2.3. Outcomes

The pre-specified primary outcome was the between-group difference in the change of total NIH-CPSI score from baseline to 12 weeks. Secondary outcomes were between-group differences in changes of total NIH-CPSI at 6 and 16 weeks, and differences in changes of pain, symptom and quality-of-life sub-scores of the NIH-CPSI at 6, 12 and 16 weeks. As safety parameters, we included voided volume, post void residual, urinalysis, and PSA at 6, 12, and 16 weeks. In addition, we used the National Cancer Institute CTCAE version 4 to categorize adverse effects with grades from 1–5 [13].

### 2.4. Ethics statement

The study was approved by the local ethics committee (Kantonale Ethikkommission Bern/3010 Bern/Switzerland/Nummer 292-07) and registered with ClinicalTrials.gov (trial registration number NCT00688506). All participants provided written informed consent before inclusion in the trial. The study conforms to the CONSORT statement (www.consort-statement.org).

### 2.5. Statistical analysis

This was a superiority trial. Assuming a standard deviation of 6 points for the NIH-CPSI total score and 10% losses to follow-up, we estimated that 30 patients per group would provide more than 80% power for an analysis of covariance adjusted for baseline values to detect a difference between groups of 4 points on the NIH-CPSI total score [14] at 12 weeks at a two-sided alpha of 0.05. Four points are associated with 90% sensitivity and 60% specificity to detect treatment response [14] and were used in previous trials to distinguish responders from non-responders [5], [14], [15]. The primary analysis was by intention-to-treat, including all randomly assigned patients in the group to which they were originally allocated to. For all continuous outcomes, we used an analysis of covariance adjusted for baseline values [16]. We then stratified the analysis of the primary outcome according to age (<50 or ≥50 years), symptom severity (≤25 or>25 points on the NIH-CPSI total score), symptom duration (≤12 or>12 months), and maximum flow rate (<15 or ≥15 mL/s), and performed formal tests of interaction between treatment and subgroup [17]. Finally, we calculated risk ratios of treatment response and corresponding numbers-needed-to-treat to achieve one treatment response defined as a decrease in 4 points on the NIH-CPSI total score. P values are two-sided. We used Stata Release 12 (StataCorp, College Station, TX) for all analyses.

## Results

### 3.1. Study participants

**Figure 1:**
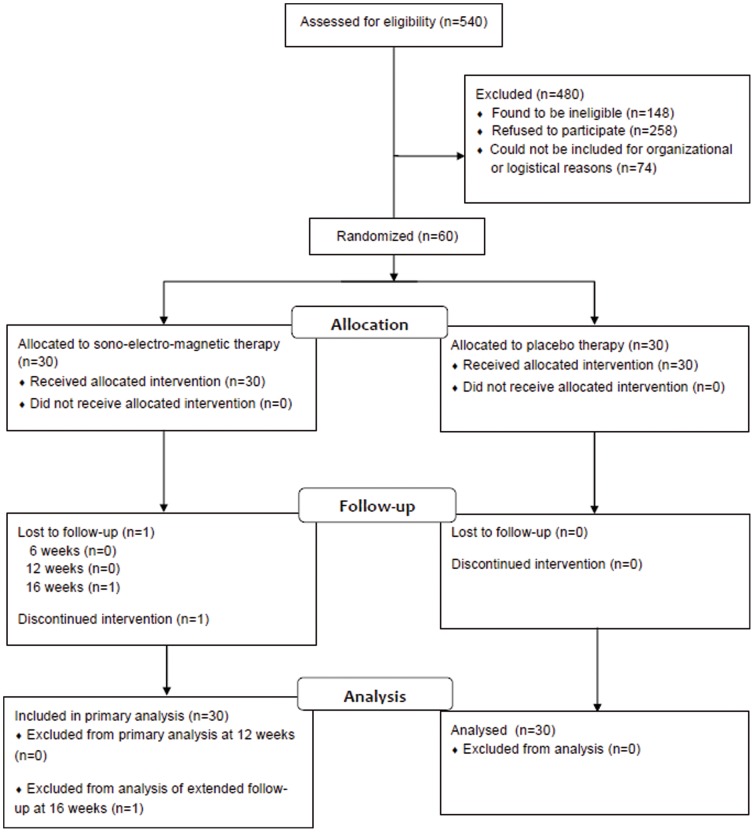
CONSORT flow diagram. Not reaching the cut-off values for study inclusion in NIH-CPSI total score was the most common reason for ineligibility. Most subjects who declined to participate considered the treatment as being too cumbersome or were not bothered enough by symptoms.

Between November 2009 and July 2012, 1342 men with potential CPPS were seen at our outpatient clinic and 540 were considered for inclusion into the trial. Of these, 148 were found to be ineligible not fulfilling the inclusion criteria, 258 refused to participate, 74 could not be included for organisational or logistical reasons. 60 eligible patients gave consent and were eventually randomized to active device (n = 30) or placebo device (n = 30). All patients completed 12 weeks of follow-up. One patient in the active group withdrew because his symptoms grew worse after 12 weeks of treatment and completed 12 weeks follow-up but did not attend the 16-week visit **(** **Fig. 1** **)**. Data on adherence to treatment was available for 45 patients. 37 patients (82%) completed 90% or more of all treatment sessions. Baseline characteristics of randomized patients are presented in **S1 Table**.

### 3.2. NIH-CPSI

**Figure 2:**
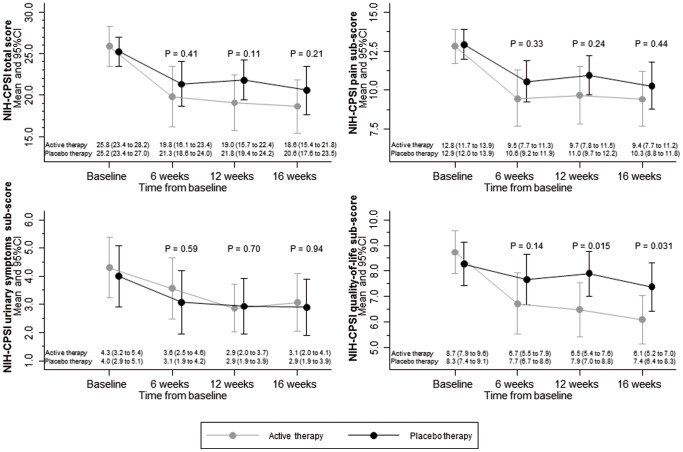
Means and 95% CI for NIH-CPSI total and sub-scores. Higher NIH-CPSI scores indicate more severe symptoms. Score ranges are as follows: total score 0 to 43; pain sub-score 0 to 21; urinary symptoms sub-score 0 to 10; quality-of-life sub-score 0 to 12. CI: confidence interval; NIH-CPSI: National Institutes of Health Chronic Prostatitis Symptom Index.

**Fig. 2** presents comparisons between groups of NIH-CPSI total and sub-scores. For the primary outcome of the NIH-CPSI total score at 12 weeks, there was a decrease from 25.8 to 19.0 with the active device and from 25.2 to 21.8 with the placebo device. Accordingly, the difference in changes was −3.1 points (95% CI −6.8 to 0.6, p = 0.11). In the analysis of treatment responders at 12 weeks, we found 21 responders in the active group (70%) and 15 responders in the placebo group (50%) who experienced a clinically relevant decrease of at least four points on the NIH-CPSI total score (risk ratio 1.40, 95% CI 0.91 to 2.15, p = 0.11). The corresponding NNT was 5 (95% CI NNT 2 to NNH 23).

In secondary comparisons of NIH-CPSI sub-scores, we found differences between groups most pronounced for the quality-of-life sub-score (difference at 12 weeks −1.6, 95%-CI −2.8 to −0.4, p = 0.015), followed by the pain sub-score (difference at 12 weeks −1.2, 95%-CI −3.3 to 0.8, p = 0.24). For the urinary symptoms sub-score, we found little evidence for a difference between groups (difference at 12 weeks −0.2, 95%-CI −1.3 to 0.9, p = 0.70 (**Fig. 2**).

**Figure 3:**
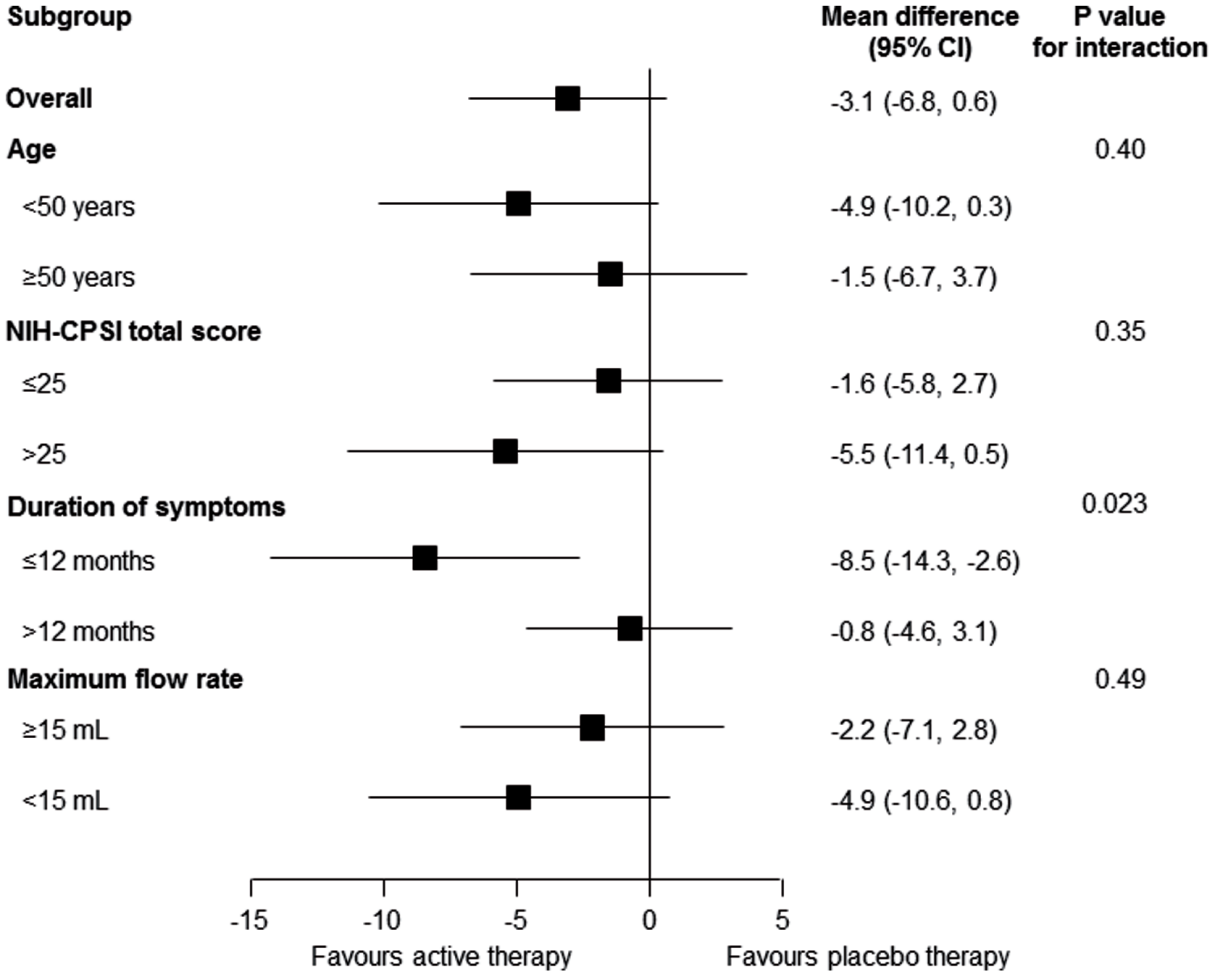
Stratified analysis according to patient characteristics. Stratified analysis of the primary outcome (NIH-CPSI total score, range 0 to 43) according to patient characteristics. CI: confidence interval; NIH-CPSI: National Institutes of Health Chronic Prostatitis Symptom Index.

**Fig. 3** presents results from stratified analyses of the primary outcome at 12 weeks according to patient characteristics. Effects varied across most subgroups to an extent compatible with chance variation. However, the benefit of the active device appeared more pronounced in patients who had a symptom duration of 12 months or less (difference between active and placebo device −8.3, 95%-CI −14.5 to 2.6) than in patients with a longer duration of symptoms (difference −0.8, 95%-CI −4.6 to 3.1; p = 0.023 for interaction between treatment and subgroup).

### 3.3. Safety parameters and adverse events

Differences in maximum flow rate, voided volume, post void residual, and PSA are presented in **S2 Table**. One out of 13 analyses yielded a significant difference at p = 0.038, with a somewhat lower post void residual in patients allocated to the active device at 6 weeks. One patient in the active group reported a worsening of symptoms at 12 weeks (3.3%), no other adverse events were reported.

## Discussion

### 4.1. Main findings

In this randomized trial in men with treatment refractory CPPS, the decrease in NIH-CPSI total score was more pronounced with sono-electro-magnetic therapy than with placebo. The observed difference of 3 points corresponds to an effect size of 0.5 to 0.6 standard deviations and is likely to be clinically relevant [18] despite its lack of statistical significance. The notion of clinical relevance is also reflected by the calculated NNT of 5, which is certainly important in view of the lack of effective therapeutic alternatives. For the quality-of-life sub-score as one of our pre-specified secondary outcomes, between-group differences formally reached statistical significance at 12 weeks (p = 0.01). In addition, the benefit of sono-electro-magnetic therapy was significantly (p = 0.023) more pronounced among patients with short symptom duration, i.e. ≤12 months.

### 4.2. Findings in the context of existing evidence

According to the gate control theory, there is a gateway in the dorsal horn of the spinal cord controlling and/or regulating the flow of nociceptive information. If appropriately stimulated, it may result in a reduction of pain perception [19]. Additional pain reduction may result from the promotion of endorphin release in the brain via neuromodulation [20] and other mechanisms [21].

Growing evidence supports a role of the central nervous system during the initiation and maintenance of pain perception in the absence of distinct peripheral triggers. Chronic pelvic pain may leave specific imprints in the brain, which in turn might predispose to CPPS [22], [23]. Spinal cord stimulation is frequently used to deliver electrical signals to the spinal cord by electrodes in the epidural space to stimulate nerve fibers. Peripheral neuromodulation is less frequently used, but is likely to recruit a larger number of nerve fibers due to a wider activation of inhibitory interneurons [24].

Electroacupuncture was found to be superior to sham electroacupuncture or exercise alone in a small randomized trial [25]. Although sacral neuromodulation [26] is a widely accepted therapy for patients with refractory non-obstructive chronic urinary retention, urgency-frequency syndrome and urgency incontinence, the value as a treatment for CPPS is unclear [27]. Nevertheless, a retrospective analysis suggested that sacral neuromodulation is more effective in patients with a short duration of symptoms [28], which is in line with our findings of more beneficial results for sono-electro-magnetic therapy in patients with a symptom duration of ≤12 months. Finally, in a small single-blind, crossover trial, invasive pudendal neuromodulation was more effective than sacral neuromodulation for treating patients with CPPS [29].

### 4.3. Limitations

The major limitation of our trial is a relative small sample size, which led to limited power in detecting between group differences smaller than 4 points on the NIH-CPSI total score. Moreover, including only patients with refractory CPPS may induce a negative selection bias. Indeed, the treatment effect may be underestimated in the present study and is probably more pronounced in patients with treatment-naive CPPS. The findings of the secondary analyses should be interpreted cautiously due to their exploratory nature, especially in view of multiple testing and the limited sample size of our trial. Assessment of therapy compliance was patient self-reported in an unmonitored setting and data on compliance was only available for 45 out of 60 included patients, which represents another limitation of this study. In addition, our results in men cannot be generalized to women with CPPS. Strengths include concealment of allocation and strict blinding of patients through the use of coded devices of identical appearance and an intention to treat analysis.

### 4.4. Implications for research

Our results indicate that sono-electro-magnetic therapy may be superior with a clinically relevant difference when compared to placebo intervention, even though, as it is expected in such patient population and trial design, placebo effects were important [30]. A larger trial of similar design is required to confirm or refute our preliminary results, particularly in patients with symptom duration of 12 months or less. If our results can be confirmed, a pilot trial in women is warranted in addition.

There are as yet no treatment standards for neuromodulation therapy, and no guidelines for therapeutic and maintenance regimens, and these must be developed through further research and testing. The stimulation parameters of sono-electro-magnetic therapy should be explored because individual adjustment of the stimulation parameters may improve the response rate, as it has for sacral neuromodulation. It is also unclear if maintenance-therapy is required to maintain the gains of treatment in CPPS.

CPPS may modulate spinal cord reflexes and brain networks via peripheral afferents, but we do not fully understand the mechanisms engaged in initiating and maintaining CPPS. Clarification of the precise mechanisms of action is needed. Direct comparisons of sono-electro-magnetic therapy to percutaneous tibial nerve stimulation and transcutaneous nerve stimulation, as well as to more invasive neuromodulation procedures such as sacral and pudendal neuromodulation, will help us understand how CPPS affects body systems. Conducting neuroimaging studies in patients who undergo CPPS may provide insight into pain processing mechanisms and eventually answer fundamental questions about neuroplasticity and the potential reversibility of conditions like CPPS.

### 4.5. Implications for practice

If our results can be confirmed in larger trials, sono-electro-magnetic therapy would offer a simple, non-invasive, widely available, inexpensive and effective approach towards treating patients with treatment refractory CPPS. The significant interaction with duration of symptoms suggests that patients should be treated early in the course of the disease.

### 4.6. Conclusions

Sono-electro-magnetic therapy did not result in a significant improvement of symptoms in the overall cohort of treatment refractory CPPS patients compared to placebo treatment. Subgroup analysis indicates, however, that patients with a symptom-duration of 12 months or less may benefit from sono-electro-magnetic therapy, warranting larger randomized controlled trials in this subpopulation.

## Supporting Information

S1 Table **Baseline characteristics of randomized patients.** Higher NIH-CPSI scores indicate more severe symptoms. Score ranges are as follows: total score 0 to 43; pain sub-score 0 to 21; urinary symptoms sub-score 0 to 10; quality-of-life sub-score 0 to 12. SD: standard deviation; NIH-CPSI: National Institutes of Health Chronic Prostatitis Symptom Index.
(DOCX)

S2 Table **Safety parameters group-specific values and between-group differences.** Safety parameters of the active and placebo therapy were similar. CI: confidence interval.
(DOCX)

S1 Database **Sonodyne Database.**
(XLS)

S1 CONSORT Checklist **CONSORT Checklist.**
(DOCX)

S1 Protocol **Trial Protocol.**
(PDF)
