# Supplementary material for: Sono-Electro-Magnetic Therapy for Treating Chronic Pelvic Pain Syndrome in Men: A Randomized, Placebo-Controlled, Double-Blind Trial
Source: PLoS One. 2014 Dec 29;9(12):e113368. doi: 10.1371/journal.pone.0113368 (PMC4278671; doi:10.1371/journal.pone.0113368)
Supplement: S1 Table — Baseline characteristics of randomized patients. Higher NIH-CPSI scores indicate more severe symptoms. Score ranges are as follows: total score 0 to 43; pain sub-score 0 to 21; urinary symptoms sub-score 0 to 10; quality-of-life sub-score 0 to 12. SD: standard deviation; NIH-CPSI: National Institutes of Health Chronic Prostatitis Symptom Index. (DOCX) [file pone.0113368.s001.docx]

**Table 1. Baseline characteristics of randomized patients**

|  | Active therapy (n=30) | Placebo therapy (n=30) |
| --- | --- | --- |
| Characteristics | mean (SD) | mean (SD) |
|  |  |  |
| Age (years) | 49.0 (14.2) | 44.9 (15.9) |
|  |  |  |
| NIH-CPSI total score | 25.8 (6.4) | 25.2 (4.7) |
| NIH-CPSI pain sub-score | 12.8 (3.0) | 12.9 (2.6) |
| NIH-CPSI urinary symptoms sub-score | 4.3 (2.9) | 4.0 (2.9) |
| NIH-CPSI quality-of-life sub-score | 8.7 (2.2) | 8.3 (2.3) |
|  |  |  |
| Duration of symptoms (months) | 43.3 (79.3) | 41.7 (51.2) |
|  |  |  |
| Maximum flow rate (mL/s) | 19 (8) | 21 (11) |
|  |  |  |
| Voided volume (mL) | 301 (141) | 287 (216) |
|  |  |  |
| Post void residual (mL) | 28 (26) | 16 (19) |
|  |  |  |
| PSA (μg/L) | 1.2 (1.0) | 0.9 (0.8) |
