# Supplementary material for: Sono-Electro-Magnetic Therapy for Treating Chronic Pelvic Pain Syndrome in Men: A Randomized, Placebo-Controlled, Double-Blind Trial
Source: PLoS One. 2014 Dec 29;9(12):e113368. doi: 10.1371/journal.pone.0113368 (PMC4278671; doi:10.1371/journal.pone.0113368)
Supplement: S2 Table — Safety parameters group-specific values and between-group differences. Safety parameters of the active and placebo therapy were similar. CI: confidence interval. (DOCX) [file pone.0113368.s002.docx]

**Table 2. Safety parameters**

|  | Active therapy  (n=30) | Placebo therapy  (n=30) | Difference | P value |
| --- | --- | --- | --- | --- |
|  |  | Mean (95% CI) |  |  |
| **Maximum flow rate (mL/s)** | |  |  |  |
| 6 weeks | 18.8 (15.4 to 22.1) | 21.6 (16.9 to 26.3) | -0.9 (-3.1 to 1.3) | 0.42 |
| 12 weeks | 19.1 (15.6 to 22.6) | 21.0 (18.3 to 23.7) | -0.9 (-4.0 to 2.3) | 0.60 |
| 16 weeks | 20.0 (16.9 to 23.1) | 21.1 (17.7 to 24.6) | -0.2 (-3.5 to 3.1) | 0.90 |
| **Voided volume (mL)** | |  |  |  |
| 6 weeks | 294 (235 to 353) | 264 (187 to 341) | 20 (-44 to 83) | 0.55 |
| 12 weeks | 338 (264 to 412) | 348 (273 to 423) | -21 (-111 to 69) | 0.65 |
| 16 weeks | 351 (277 to 424) | 365 (278 to 452) | -32 (-129 to 65) | 0.52 |
| **Post void residual (mL)** | |  |  |  |
| 6 weeks | 10.6 (5.7 to 15.5) | 23.6 (8.1 to 39.1) | -17.1 (-32.9 to -1.4) | 0.038 |
| 12 weeks | 20.5 (10.8 to 30.2) | 25.6 (1.1 to 50.1) | -12.8 (-38.0 to 12.5) | 0.33 |
| 16 weeks | 14.8 (7.2 to 22.4) | 25.2 (1.4 to 49.0) | -19.8 (-44.3 to 4.7) | 0.12 |
| **PSA (μg/L)** |  |  |  |  |
| 6 weeks | 1.2 (0.9 to 1.6) | 0.9 (0.6 to 1.2) | 0.1 (-0.2 to 0.3) | 0.61 |
| 12 weeks | 1.6 (0.7 to 2.5) | 0.9 (0.6 to 1.2) | 0.5 (-0.3 to 1.3) | 0.25 |
| 16 weeks | 1.3 (0.9 to 1.7) | 0.9 (0.6 to 1.2) | 0.2 (-0.2 to 0.5) | 0.31 |
