# Supplementary material for: Sono-Electro-Magnetic Therapy for Treating Chronic Pelvic Pain Syndrome in Men: A Randomized, Placebo-Controlled, Double-Blind Trial
Source: PLoS One. 2014 Dec 29;9(12):e113368. doi: 10.1371/journal.pone.0113368 (PMC4278671; doi:10.1371/journal.pone.0113368)
Supplement: S1 Protocol — Trial Protocol. (PDF) [file pone.0113368.s005.pdf]

Sonodyn® Medico Star for treating chronic pelvic pain syndrome in men: A randomized, placebo-controlled, double-blind trial

Summary

Background

Chronic pelvic pain syndrome (CPPS) represents besides benign prostatic hyperplasia (BPH) and prostate cancer the third most common diagnosis in men with prostatic complaints under the age of 50 years [1]. Thus, CPPS is a serious economic burden on any health care system and quality of life is significantly limited in affected individuals.

Treatment of CPPS is challenging for patients and physicians once conventional therapies fail. A large number of therapeutic approaches have been propagated, with limited effectiveness, however, and the ideal therapy remains to be elucidated.

Our Sonodyn® Medico Star pilot study that treated patients using a combination of different kinds of neuromodulation reported encouraging findings: Sono-electro-magnetic therapy achieved an improvement in more than 40% of patients with refractory CPPS. These results are promising especially considering the negative patient selection.

Purpose

We hypothesize that combined sono-electro-magnetic therapy can improve refractory CPPS in men. In addition, we postulate that combined sono-electro-magnetic therapy as well as placebo therapy has a significant effect on brain activity detectable by functional MRI.

Study end-points

Primary outcome measures: National Institutes of Health Chronic Prostatitis Symptom Index (NIH-CPSI) (time frame: before treatment, after 6 and 12 weeks of treatment as well as 4 weeks after stop of treatment)

Treatment success is defined as an improvement of at least 4 points in the NIH-CPSI total score or a reduction in the NIH-CPSI pain score to a value < 8.

Secondary outcome measures: Brain activity assessed by functional MRI (time frame: before treatment, after 12 weeks of treatment)

Comparison of MRI of CPPS patients versus MRI of age-/sex-matched controls of the local neuro-radiology MRI database (time frame: before treatment)

Tertiary outcome measures: Side effects.

Study design

Prospective, randomized, double-blind, placebo-controlled study.

Setting: Outpatient clinic, Department of Urology and Neuro-Radiology, Inselspital Bern

Study population

62 patients.

Inclusion criteria

CPPS III, duration of symptoms >3 months, NIH-CPSI total score ≥15, NIH-CPSI pain score ≥8, status post antibiotic therapy with a tetracycline for 4 weeks, status post α-blocker treatment for at least 6 weeks, status post non-steroidal anti-inflammatory drug therapy. Written informed consent.

Exclusion criteria

Inclusion criteria not fulfilled. Post-void residual >100ml, nitrite-positive urine sample, positive urine culture (Meares-Stamey 3-glass test and post-prostatic massage urine). Urethral stricture, prostate cancer, age <18 years, claustrophobia, cardiac pacemaker, implanted nerve-stimulator, insulin or pain pump.

Interventions

Meares-Stamey 3-glass test, uroflowmetry, measurement of post-void residual, measurement of PSA

NIH-CPSI and pain diary

Treatment with Sonodyn® Medico Star therapy over a treatment period of 12-weeks (depending on the randomisation sono-electro-magnetic treatment or placebo treatment)

MRI of the brain: before therapy and 12 weeks after therapy.

Study flowchart

Study candidate

End of the trial: unblinding

Therapy with verum device
Therapy and pain diary

Therapy with placebo device
Therapy and pain diary

PSA measurement
Urinalysis and urine culture
Uroflowmetry and post-void residual measurement
NIH-CPSI

Signature page

Investigators

Pract. med. Katharina Haeni
Resident
Department of Urology
Inselspital
3010 Bern

Dr. med. Mirjam Huwyler
Junior staff member
Department of Urology
Inselspital
3010 Bern

Dr. med. Thomas Kessler
Junior staff member
Department of Urology
Inselspital
3010 Bern
and scientific co-worker Horten-Zentrum
Universitiy Hospital Zürich
8091 Zürich

Dr. med. Roland Wiest
Staff member
Department of Neuro-Radiology
Inselspital
3010 Bern

Prof. Dr. med. George Thalmann
Chairman
Department of Urology
Inselspital
3010 Bern

Bern, 17.12.2007

Background

Chronic pelvic pain syndrome (CPPS) represents besides benign prostatic hyperplasia (BPH) and prostate cancer the third most common diagnosis in men with prostatic complaints under the age of 50 years [1]. Approximately 8% of all urological outpatient consultations are due to CPPS [2]. Thus, CPPS is a serious economic burden on any health care system and quality of life is significantly impaired in affected individuals.

The exact patho-mechanisms involved in CPPS are still unknown and many factors seem to contribute to this heterogeneous disorder [3]. Multimodal therapies are more successful than single therapies and a combination of antibiotics, alpha-blockers and/or nonsteroidal anti-inflammatory drugs seem to help some patients. However, approximately 30% to 50% of all patients find no relief in conventional therapies.

Our Sonodyn® Medico Star pilot study that treated patients using a combination of different kinds of neuromodulation reported encouraging findings: Sono-electro-magnetic therapy achieved an improvement in more than 40% of patients with treatment refractory CPPS after a treatment period of 6 and 12 weeks, respectively [4].

These results are promising especially considering the negative patient selection with various unsuccessful conventional therapies prior to sono-electro-magnetic treatment.

The mechanism of action of Sonodyn® Medico Star treatment in men with refractory CPPS is largely unknown. However, a neuromodulative effect on different brain centers seems to be involved. A placebo-controlled trial setting is, in contrast to other neuro-modulative techniques, such as transcutaneous electric nerve stimulation (TENS) or sacral neuromodulation (SNM), possible with Sonodyn® Medico Star as patients cannot perceive the device working because it uses subsensory stimulation.

Purpose

We hypothesize that combined sono-electro-magnetic therapy can improve refractory CPPS in men. In addition, we postulate that combined sono-electro-magnetic therapy as well as placebo therapy has a significant effect on brain activity detectable by functional MRI.

Hypothesis

Based on our pilot study we assume, that a 12-week treatment course with sono-electro-magnetic therapy will significantly improve symptoms in men with refractory CPPS compared to placebo treatment.

Furthermore we assume a neuromodulative effect of the sono-electro-magnetic therapy on different brain centers.

Study end-points

Primary outcome measures: National Institutes of Health Chronic Prostatitis Symptom Index (NIH-CPSI) (time frame: before treatment, after 6 and 12 weeks of treatment as well as 4 weeks after stop of treatment)

Treatment success is defined as an improvement of at least 4 points in the NIH-CPSI total score or a reduction in the NIH-CPSI pain score to a value < 8.

Secondary outcome measures: Brain activity assessed by functional MRI (time frame: before treatment, after 12 weeks of treatment)

Comparison of MRI of CPPS patients versus MRI of age-/sex-matched controls of the local neuro-radiology MRI database (time frame: before treatment)

Tertiary outcome measures: Side effects.

Study design

Prospective, randomized, double-blinded, placebo-controlled study.

Setting: Outpatient clinic Department of Urology and Neuro-Radiology, Inselspital Bern

Study population

62 patients

Inclusion criteria

- CPPS III
- Duration of symptoms >3 months
- NIH-CPSI total score ≥ 15
- NIH-CPSI pain score ≥ 8,
- Status post antibiotic therapy with a tetracycline for 4 weeks
- Status post α-blocker treatment for at least 6 weeks
- Status post non-steroidal anti-inflammatory drug therapy
- Written informed consent

Exclusion criteria

- Inclusion criteria not fulfilled
- Post-void residual of >100mL
- Nitrite-positive urine sample
- Positive urine culture (Meares-Stamey 3-glass test and/or post-prostatic massage urine)
- Urethral stricture
- Prostate cancer
- Age <18 years
- Claustrophobia
- Cardiac pacemaker, implanted nerve stimulator, insulin or pain pump

Rules for stopping and unblinding of the trial ahead of schedule

- Aggravation of symptoms under therapy

Interventions

Before study inclusion: Meares-Stamey 3-glass test, uroflowmetry, measurement of post-void residual, measurement of PSA, NIH-CPSI
Study inclusion: Handover of the treatment device.
Patients are instructed on how to perform sono-electro-magnetic therapy at home and to apply the device on the perineum daily in the morning and evening for the duration of ten minutes each time over the time period of 12 weeks. During the whole treatment period patients have to document for every single day in a diary whether they performed the treatment and they also have to conduct a pain diary. In addition, potential co-medication has to be documented in the dairy.
MRI of the brain is performed before initiation of sono-electro-magnetic therapy.

After 6 and 12 weeks of therapy**:** Urinalysis and urine culture, uroflowmetry, post-void residual measurement and PSA measurement, NIH-CPSI.

After 12 weeks of therapy: MRI of the brain and stop of therapy

16 weeks after initiation of therapy: Urinalysis, uroflowmetry, measurement of post-void residual, measurement of PSA, NIH-CPSI

Study end and unblinding: Patients with insufficient treatment response after placebo-therapy will receive a verum Sonodyn device by the manufacturer without additional cost for another 12 weeks of treatment.
Patients with insufficient treatment response under verum therapy will be reassessed in the urological outpatient clinic and further treatment options will be considered, for instance physiotherapy.

Statistical analyses: sample size and power calculation

To detect a 4 point difference in the NIH-CPSI total score between baseline and after 12 weeks of therapy (assuming a standard deviation of 6.8, based on the data of the pilot study) at a two-sided

significance level of 5% (α=0.05) with a power of 80% (β=0.20) a total of 50 patients has to be recruited (i.e. 25 patients in the verum and 25 patients in the placebo group, respectively). Considering a drop-out rate of 20%, a total of 62 patients should be included into the trial.

Risks and inconveniences

Up to now, there are no known risks or side effects by external application of Sonodyn® Medico Star for treating muscle and joint complaints.

In our pilot study in men with treatment refractory CPPS, there was 1 patient complaining about aggravation of symptoms under sono-electro-magnetic therapy and no other side effects were documented [4]. It is unclear, however, whether the symptom aggravation was really due to sono-electro-magnetic therapy or whether it was just the spontaneous course of the disease. Patients with contraindications for MRI of the brain are excluded from the trial.

Verum and placebo device

Name: Sonodyn® Medico Star

Manufacturer: Sonodyn AG, Schöngrünstr. 27, 4500 Solothurn
Tel.: +41 32 628 60 00, Fax: +41 32 628 60 09, Email: info@sonodyn-ag.ch

Patients will be randomly allocated to sono-electro-magnetic or placebo therapy based on computer-generated random numbers. The manufacturer prepares, pre-packs and sequentially numbers the active and placebo devices according to the concealed randomization schedule.

Sonodyn Corporation AG (Solothurn, Switzerland) will kindly provide all stimulation devices.

References

[1] Roberts RO, Lieber MM, Bostwick DG, Jacobsen SJ. A review of clinical and pathological prostatitis syndromes. Urology 1997; 49:809-21.

[2] Collins MM, Stafford RS, O'Leary MP, Barry MJ. How common is prostatitis? A national survey of physician visits. J Urol 1998; 159:1224-8.

[3] McNaughton Collins M, MacDonald R, Wilt TJ. Diagnosis and treatment of chronic abacterial prostatitis: a systematic review. Ann Intern Med 2000; 133:367-81.

[4] Kessler TM, Z'Brun S, Thalmann GN. Kombinierte sono-elektro-magnetische Therapie: Neue Hoffnung bei chronischem Schmerzsyndrom des Beckens? 63. Jahrestagung der Schweizerischen Gesellschaft für Urologie, September 2007.
